# MultiDMPcaller: a one-stop software for detection and visualization of differentially methylated positions and regions

**DOI:** 10.1093/bioinformatics/btag655

**Published:** 2026-08-31

**Authors:** Qiuyu Yuan, Hongyan Zhao, Zhijun Zhang, Chenhao Yue, Bingchao Zhang, Songyan Xue, Qing Zou, Jiantao Yu

**Affiliations:** College of Information Engineering, Northwest A&F University, Yangling 712100, China; College of Information Engineering, Northwest A&F University, Yangling 712100, China; College of Information Engineering, Northwest A&F University, Yangling 712100, China; College of Information Engineering, Northwest A&F University, Yangling 712100, China; College of Information Engineering, Northwest A&F University, Yangling 712100, China; College of Information Engineering, Northwest A&F University, Yangling 712100, China; College of Information Engineering, Northwest A&F University, Yangling 712100, China; College of Information Engineering, Northwest A&F University, Yangling 712100, China

## Abstract

**Motivation:**

Whole-genome bisulfite sequencing (WGBS/BS-Seq) is the gold standard for single-base resolution DNA methylome profiling. However, the diverse statistical models of existing computational methods lead to limited overlap between their results, highlighting the need for novel methods to detect differentially methylated positions (DMPs) and differentially methylated regions (DMRs).

**Results:**

We developed MultiDMPcaller, an automated downstream methylome analysis software. It processes upstream outputs to profile DMPs, non-DMPs, DMRs, and context-specific (CpG/CHG/CHH) methylation status, alongside visualizing their chromosomal distribution and enrichment. The software features two key innovations: (i) an adaptive two-step *P*-value adjustment strategy based on organism-specific methylation patterns, with raw *P*-value ≤0.05 pre-filtering followed by false discovery rate (FDR) correction, to recover potential DMPs usually missed by standard FDR correction in plant CHG/CHH and animal CpG contexts; and (ii) a multiple pairwise comparison approach, which performs *m *×* n* pairwise comparisons for *m* control and *n* experimental replicates, followed by a voting system supporting both user-defined majority thresholds and model-based adaptive thresholds, to identify robust and reliable DMPs (with a stricter voting threshold exclusively for loci with low methylation differences) and DMRs. On real datasets from *Arabidopsis*, apple, and mouse, as well as simulated human datasets, MultiDMPcaller’s results showed good agreement with those of other software, exhibiting high conservativeness and superior precision, which suggested a low false discovery proportion.

**Availability and implementation:**

MultiDMPcaller is available at GitHub (https://github.com/jiantaoyuNWAFU/MultiDMPcaller) and via a web server (https://ciebioinfo.nwafu.edu.cn).

## 1 Introduction

As one of the core epigenetic modifications, DNA methylation is profiled genome-wide at single-base resolution via whole-genome bisulfite sequencing (WGBS). Researchers routinely compare methylation patterns between different biological conditions (e.g. control vs. experimental groups) to identify statistically significant differentially methylated positions (DMPs) and differentially methylated regions (DMRs), which are critical for dissecting epigenetic regulatory mechanisms.

To mitigate noise from random fluctuations across biological replicates and identify robust, significant DMPs and DMRs, researchers widely apply statistical frameworks tailored for multi-group, multi-replicate methylome data. Commonly used models include logistic regression, beta regression, analysis of variance, beta-binomial, and classical Bayesian models, implemented in mainstream tools, such as methylKit (Akalin *et al.* 2012), DMRcaller (Catoni *et al.* 2018), DSS (Feng and Wu 2019), MethylSig (Park *et al.* 2014), and DMRcate (Peters *et al.* 2021). Supporting statistical tests include the chi-square test, Wald test, likelihood ratio test, and t-test, while Fisher’s exact test is the conventional approach for single-replicate experimental designs. For DMR identification, the mainstream strategy is sliding-window prescreening with statistical filtering (e.g. MethylSig, DMRcate). Significant DMPs/DMRs are typically called at a Benjamini–Hochberg (BH) adjusted *P*-value cutoff of 0.05 and a methylation difference >25%, with hyper- and hypomethylation assigned based on the direction of change.

**Figure 1:**
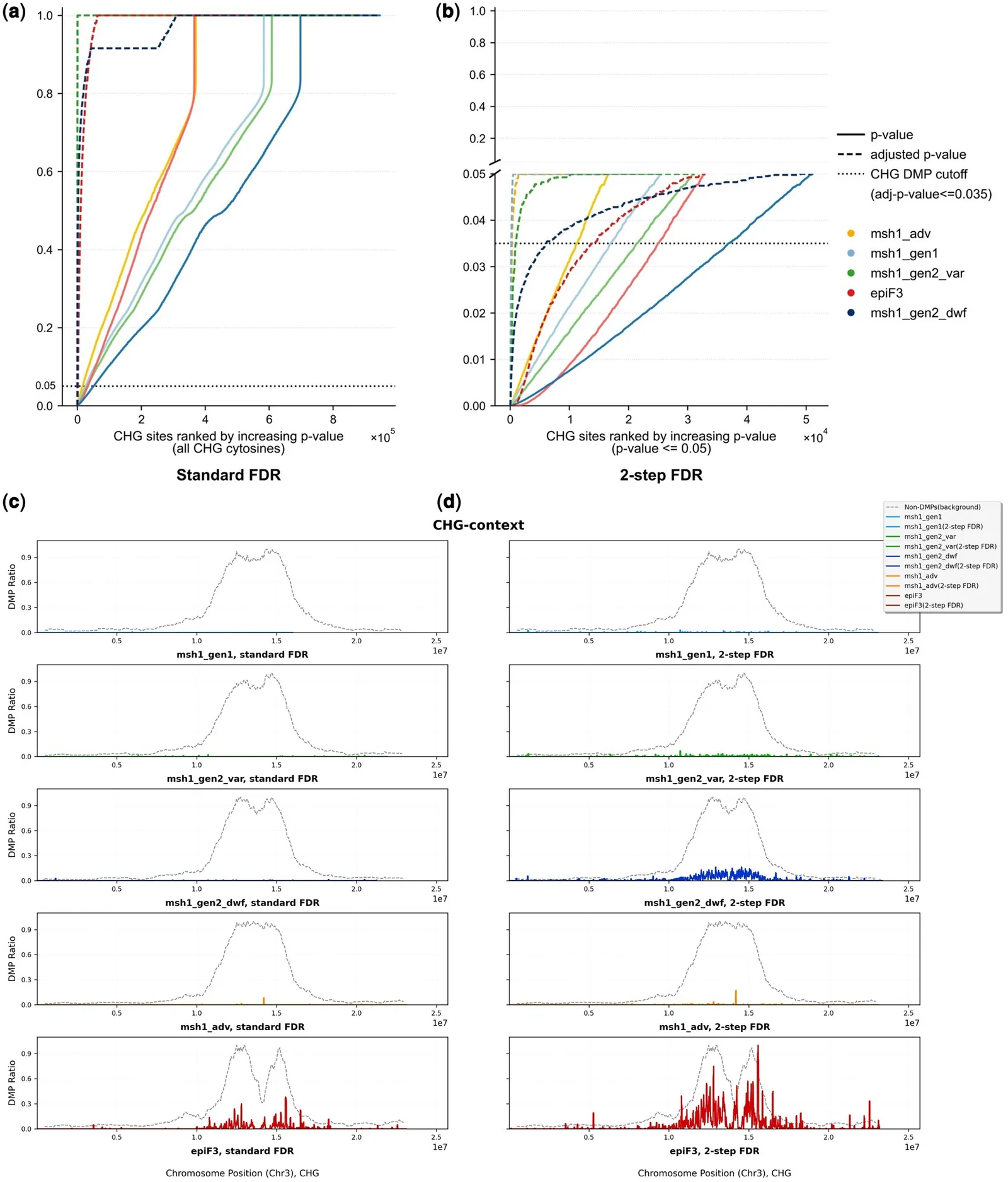
CHG-DMP detection by (a) the conventional one-step method and (b) the two-step method, with distribution profiles in (c) and (d), respectively, on *Arabidopsis thaliana* chromosome 3 (*mut1* vs wt1).

Here we present MultiDMPcaller with a two-step *P*-value adjustment for weak signal recovery and an *m *×* n* pairwise comparison and voting framework with built-in false discovery control. The workflow is in Fig. 1, available as supplementary data at *Bioinformatics* online.

## 2 Methods

### 2.1 Input module

The module accepts *m* control and *n* experimental files, each with five columns (chromosome, coordinate, methylated and unmethylated read counts, and context; Table 1, available as supplementary data at *Bioinformatics* online), and these files are easily generated from upstream tools.

**Table 1:** Data sources for Fisher’s exact test.

	Methylated read count	Unmethylated read count
Experimental sample	*a*	*b*
Control sample	*c*	*d*

### 2.2 Statistical analysis module

#### 2.2.1 Fisher’s test and false discovery rate correction

For each cytosine site, methylated (*a*/*c*) and unmethylated (*b*/*d*) read counts are extracted from one experimental and one control replicate (Table 1). Pre-filtering requires ≥2 methylated reads in either group (*a* or *c *≥ 2) and ≥4 total reads per group (*a *+* b*, *c *+* d *≥ 4) to remove ultra-low-coverage sites. Raw *P*-values are derived from Fisher’s exact test. All genome-wide cytosine *P*-values are then pooled for false discovery rate (FDR) correction to yield *q*-values, with *q *≤ 0.05 for DMPs (*q *> 0.05: non-DMPs) and *a*/(*a *+* b*) > *c*/(*c *+* d*) for hypermethylation (otherwise hypomethylation).

#### 2.2.2 Adaptive *P*-value adjustment strategy

In plants, the number of CHG and CHH methylation sites is generally much higher than that of CpG sites. Therefore, applying the same *P*-value adjustment method for CHG/CHH sites as for CpG sites results in overly stringent filtering. For example, when *P*-values of all CHG sites across chromosomes are pooled into a single set for adjustment, the larger the set size, the greater the correction factor *k*/*i* in Equation (1), leading to a steeper increase in *q*-values (Fig. 1a).


(1) $$q_{\left(i\right)}\approx p_{\left(i\right)}\times \frac{k}{i}$$

where *k* is the total number of loci in the adjustment set, and *i* denotes the rank of the *P*-value sorted in ascending order. Consequently, fewer sites meet the *q*-value threshold (typically 0.05), resulting in fewer identified DMPs (Fig. 1c). This uniform significance threshold leads to the omission of many potential sites. We define these loci as “potential” sites: as shown in Fig. 1b, at raw *P*-value ≤ 0.05, msh1_gen2_dwf and *epiF3* genotypes exhibit more discernible CHG methylation changes than other genotypes (e.g. *msh1_gen1*, *msh1_gen2_var*, *msh1_adv*), supported by their lower *P*-value trend. However, conventional genome-wide FDR correction masks these subtle differences, driving a sharp increase in *q*-values across all genotypes and resulting in consistently few DMPs in every genotype. The same pattern is observed for CHH contexts (Fig. 2a–c, available as supplementary data at *Bioinformatics* online).

For CHG and CHH methylation sites in plants, we adopt the following two-step *P*-value adjustment strategy, referred to as the two-step FDR method. First, sites with raw *P*-value ≤0.05 are selected to form a subset. Second, *P*-value to *q*-value adjustment is performed on this subset. Compared with conventional FDR correction, the increase in *q*-values is much more moderate, which facilitates the detection of subtle methylation changes (Fig. 1b, Fig. 2b, available as supplementary data at *Bioinformatics* online). Finally, sites with *q*-value ≤0.035 (for CHG) and *q*-value ≤0.049 (for CHH) are selected as significant DMPs. The thresholds of 0.035 and 0.049 are manually specified, as these cutoffs help to reveal differences in DMP counts across genotypes. In addition to manual specification, MultiDMPcaller can determine this data-driven threshold based on the maximum difference between *q*-values and raw *P*-values, termed adaptive *q*-value threshold (AQT). The validity of this adaptive threshold and its proximity to manually defined cutoffs are validated in Fig. 3, available as supplementary data at *Bioinformatics* online. With this approach, we observe a substantial increase in DMP counts for certain genotypes (e.g. CHG in *msh1_gen2_dwf*) compared with the one-step method, but no comparable increase for others (e.g. CHG in *msh1_adv*) (Fig. 1d, Fig. 2d, available as supplementary data at *Bioinformatics* online). This indicates that the two-step *P*-value adjustment strategy does not simply relax thresholds to inflate DMP numbers across all genotypes; instead, it retains some “promising” sites via prefiltering to capture those subtle methylation differences between genotypes.

The two-step adjustment is applied only to animal CpG sites (the dominant methylation context), with conventional FDR for CHG and CHH. All contexts in other organisms use conventional correction.

It should be noted that our two-step *P*-value adjustment is an empirical heuristic approach. It does not satisfy the independent filtering assumption (Bourgon *et al.* 2010)—which requires the screening criterion to be independent of the test statistic under the null hypothesis—since pre-filtered *P*-values are reused in BH correction. However, it performs well in practice for capturing subtle methylation signals. We compared our two-step method with an independence-compliant approach (removing low-coverage sites prior to BH correction) and found that the two-step method achieved higher recall while maintaining well-controlled false discovery (Fig. 4, available as supplementary data at *Bioinformatics* online).

To address the inherent risk of elevated false positives associated with this two-step strategy, we implement a suite of complementary false discovery control measures: low-difference strict voting (LowDiff-StrictVote), AQT, and adaptive voting threshold for DMPs (AVT-DMP). Given that the false discovery proportion (FDP) is higher among loci with small absolute methylation differences (Fig. 5, available as supplementary data at *Bioinformatics* online), we apply the LowDiff-StrictVote strategy to raise the voting threshold for these loci to enter the final DMP set (Table 2, available as supplementary data at *Bioinformatics* online). AQT and AVT-DMP further define the signal–noise boundary, facilitating reliable DMP detection.

#### 2.2.3 Integration of *m *×* n* pairwise comparison results

For *m* control and *n* experimental replicates, *m *×* n* pairwise Fisher’s exact tests and *P*-value adjustment are performed sequentially, with majority voting applied to the results: a site enters the final reliable DMP set (finalDMP) if its support count meets or exceeds the specified threshold. In addition to user-defined thresholds, we adopt the bimodal Gaussian mixture model valley thresholding approach to adaptively determine this cutoff, termed the AVT-DMP. Based on the support count distribution of candidate DMP loci across support levels from 1 to *m *×* n* pairwise comparisons, this approach identifies the voting boundary for robust DMP calls (Fig. 6, available as supplementary data at *Bioinformatics* online and Table 3, available as supplementary data at *Bioinformatics* online). In addition, the hyper-/hypomethylation status of final DMPs is determined by majority voting.

#### 2.2.4 Definition and filtering of DMRs

DMRs are regions highly enriched for DMPs. DMR identification has two steps: (i) candidate generation; and (ii) reliable filtering.

For candidate DMR identification, we refer to the method of Lister *et al.* (2009) and perform three steps on the finalDMP dataset: first, scanning the genome with a 1 kb sliding window (100 bp step) to identify DMP-dense windows (≥4 DMPs per window); second, bidirectionally extending and merging consecutive dense windows to form dense regions; and finally, merging overlapping or consecutive dense regions via the widest boundary principle, and retaining regions ≥1 kb in length with ≥10 DMPs as candidate DMRs.

For each candidate DMR, methylated and unmethylated read counts are aggregated across all sites within the region for Fisher’s exact test and subsequent BH FDR correction (*q *≤ 0.05). This procedure is repeated for all *m *×* n* pairwise comparisons. The final DMR set is determined via either user-defined thresholds or the adaptive voting threshold for DMRs (AVT-DMR, same algorithm as AVT-DMP) (Fig. 7, available as supplementary data at *Bioinformatics* online), with hyper-/hypomethylation status assigned by majority voting.

### 2.3 Visualization module

As shown in Fig. 8, available as supplementary data at *Bioinformatics* online, we use a 1000 kb sliding window (50 kb step size) to quantify DMPs within each window. The DMP enrichment level of each window is normalized to *n*/*maxd*, where *n* is the DMP count of the window, and *maxd* is the global maximum DMP count across all chromosomes. This normalization method enables straightforward cross-chromosome comparison of differential methylation levels. Hypermethylation, hypomethylation, and non-DMP profiles are also plotted alongside the overall DMP curve. Each DMR is marked by a short vertical line (green for hyper-DMRs, blue for hypo-DMRs) above the DMP curve. Additionally, functional descriptions of all software options are provided in Table 4, available as supplementary data at *Bioinformatics* online.

## 3 Results

We performed a reanalysis of the *Arabidopsis thaliana* methylome datasets from Virdi *et al.* (2015). Data quality and DMP/DMR overviews are provided in Fig. 9, available as supplementary data at *Bioinformatics* online and Tables 5–6, available as supplementary data at *Bioinformatics* online. We selected epiF3 samples with prominent methylation changes for comparison between MultiDMPcaller and other mainstream tools. We generated and analyzed three human simulated methylome datasets with replicate designs of 2 × 2, 3 × 3, and 4 × 4 using pWGBSSimla (Chung and Kang 2020) (Fig. 10, available as supplementary data at *Bioinformatics* online). We further analyzed public methylome datasets from apple and mouse (Xu *et al.* 2018, Niu *et al.* 2019, He *et al.* 2020). Tool configurations, computational performance, and scalability are detailed in Tables 7–9, available as supplementary data at *Bioinformatics* online.

Compared with the conventional one-step FDR method, our two-step adjustment strategy in MultiDMPcaller identified additional DMPs, and this advantage was particularly pronounced in real biological data. The two-step DMP set encompassed the vast majority (83%–100%) of one-step DMPs, with total counts 3.6/3.8 times those of the one-step method for *Arabidopsis* CHG/CHH, 1.2/2.5 times for apple CHG/CHH, and 1.6 times for mouse CpG contexts (Figs 11 and 12, available as supplementary data at *Bioinformatics* online). A critical question is whether these additional DMPs come with inflated false positives. MultiDMPcaller addressed this via LowDiff-StrictVote, AQT, and AVT-DMP. With these strategies, the two-step method reduced the FDP by ∼13.8 percentage points relative to the one-step method on human simulated datasets (Fig. 11d, available as supplementary data at *Bioinformatics* online). For real datasets (*Arabidopsis*, apple, and mouse), where exact FDP cannot be directly quantified, we used the overlap rate with established tools as an indirect indicator of false discovery control. Across most datasets, 97%–100% of two-step DMPs were covered by existing tools; the only outlier was apple CHH, with 71% overlap. This demonstrates high conservatism of our approach (Figs 11 and 13, available as supplementary data at *Bioinformatics* online).

To further verify the false discovery control performance of this combined pipeline, we compared two-step adjustment outputs with and without these strategies using *Arabidopsis* data. Without the strategies, MultiDMPcaller DMP calls showed low overlap with existing tools, with many unique calls likely harboring false positives. DMP overlap rates were 24.7%–53.9% across all three contexts (Figs 14 and 15, available as supplementary data at *Bioinformatics* online). Regardless of divergent DMR definitions across tools, CpG- and CHG-DMR overlap was 40.0%–100%, whereas CHH-DMR overlap remained very low due to sparse counts (Fig. 16a and b, available as supplementary data at *Bioinformatics* online).

By contrast, after applying these strategies, 97%–100% of DMPs identified by MultiDMPcaller were covered by other tools (Fig. 11j, available as supplementary data at *Bioinformatics* online). This shift was driven primarily by AQT—slightly stricter than the manually specified cutoff—which reduced baseline CHG and CHH DMP counts by 25.7% and 8.6%, respectively; the LowDiff-StrictVote strategy made a secondary contribution (4.1% and 6.2% reductions for CHG and CHH). DMR counts decreased slightly, while overlap with other tools remained largely unchanged (Fig. 16c, available as supplementary data at *Bioinformatics* online). Taken together, these measures drove MultiDMPcaller calls toward higher conservatism and better false discovery control.

A similar pattern was observed in the human simulated data. After applying the LowDiff-StrictVote + AQT + AVT-DMP pipeline, MultiDMPcaller achieved higher precision (5.4%–54.3%) and slightly lower recall (1.1%–5.7%) than competing tools (methylKit, DSS, MethylSig) in most scenarios (Fig. 17, available as supplementary data at *Bioinformatics* online). Precision and recall typically show a trade-off relationship; MultiDMPcaller achieved the best performance on balanced metrics F1 and Matthews Correlation Coefficient (MCC). Notably, loci with low absolute methylation differences (0–0.3) carried a high FDP: among 2823 such loci in the 2 × 2 dataset, 2441 (∼86.47%) were false positives, and the majority of these false positives (1776 loci, ∼72.76%) only received three out of four pairwise supports rather than unanimous agreement (Fig. 5, available as supplementary data at *Bioinformatics* online). We therefore raised the voting threshold for these low-difference loci from ≥3 to unanimous support. Results showed that this strategy reduced the FDP in the low-difference range by ∼14.5 percentage points (Fig. 5, available as supplementary data at *Bioinformatics* online).

In addition, in *Arabidopsis*, the DMPs detected by MultiDMPcaller correlated strongly with differentially expressed small RNA clusters: upregulated clusters were predominantly associated with hypermethylation across all three contexts, while downregulated clusters were linked to hypomethylation, consistent with previous studies (Bond and Baulcombe 2015). We validated this relationship using a machine learning classifier that predicts cluster up/downregulation from the counts of hyper- and hypomethylated DMPs within each cluster, achieving 77.8% and 92.9% accuracy for clusters containing ≥1 and ≥3 DMPs, respectively (Table 10, available as supplementary data at *Bioinformatics* online). Comparable accuracy and the same hypermethylation-expression upregulation coupling were observed in apple (Table 11, available as supplementary data at *Bioinformatics* online), further confirming the biological relevance of MultiDMPcaller-derived DMPs.

## Supplementary Material

btag655_Supplementary_Data

## Data Availability

Public datasets: *Arabidopsis* (GEO: GSE36783), apple (SRA: SRP097376, SRP110881), mouse (GEO: GSE82945, GSE82621). Human simulated data, supplementary *Arabidopsis* data, and MultiDMPcaller code are deposited at Zenodo (DOIs: 10.5281/zenodo.21489519, 10.5281/zenodo.22109051, 10.5281/zenodo.21615926).
