# Supplementary material for: MultiDMPcaller: a one-stop software for detection and visualization of differentially methylated positions and regions
Source: Bioinformatics. 2026 Aug 31;42(9):btag655. doi: 10.1093/bioinformatics/btag655 (PMC13561517; doi:10.1093/bioinformatics/btag655)
Supplement: Supplementary file 1 [file btag655_supplementary_data.docx]

Supplementary Materials

**Supplementary Figure 1**. Analysis workflow of MultiDMPcaller

**Supplementary Figure 2**. Performance comparison between conventional FDR and two-step p-value adjustment for DMP identification: CHH DMPs on Arabidopsis chr3 (*mut*1 vs wt1).

**Note:** Left panels (a, c): P-values sorted in ascending order show that *msh1_gen2_dwf*/*msh1_gen2_var* (CHH) have smaller p-values than other genotypes, indicating more discernible methylation changes in these genotypes. However, conventional genome-wide FDR correction yields q-values ≥ 0.05 for most loci (dashed lines), filtering them out of the DMP set.

Right panels (b, d): Our proposed two-step strategy (pre-filtering loci with raw p-value ≤ 0.05 followed by FDR correction on the subset) yields a more moderate increase in q-values and captures between-genotype differences in DMP counts. An empirical q-value threshold of 0.049 (CHH) was applied to define final DMPs, retaining a large number of DMP loci masked by conventional correction; this threshold was optimized based on q-value curve trends to maximize detection of between-genotype differences. You can also use the --auto-qvalue-twostep option to obtain the adaptive q-value threshold (AQT).

The distribution was visualized using a sliding window approach (window size: 40 kb, step size: 2 kb). DMP enrichment density calculation was performed following the method described in the “Visualization Module” section of the main text.

**Supplementary Figure 3**. Determination of the adaptive q-value threshold (AQT). (a) Taking the human 3×3 simulated dataset as an example, the trend of p-values (solid lines) and q-values (dashed lines) for each single pairwise comparison. The circle marks the AQT on the y-axis, i.e., the point where the difference *Δ* = q-value − p-value is maximized. (b) Trend of the difference between q-value and p-value. The circle marks the point with the maximum difference. (c) Values of evaluation metrics when q-value thresholds are set from 0.01 to 0.05, as well as for the AQT. The AQT corresponds to near-optimal performance. (d–e) Comparison between the adaptive q-value threshold AQT (d for CHG, e for CHH) and empirically determined thresholds (0.035 for CHG, 0.049 for CHH) on the *Arabidopsis thaliana* 2×2 dataset. The circle marks the AQT.

**Note:** We generated human simulated methylome data using the pWGBSSimla method (Chung and Kang, 2020), and added an adaptive q-value threshold (AQT) determination strategy. Based on the distribution of p-values and q-values from the two-step adjustment method, this strategy selects the q-value corresponding to the point with the maximum difference between q-value and p-value (q-value − p-value) as the threshold (Supplementary Figure 3a-b). The rationale is as follows: below the threshold, the difference between p-values and q-values gradually increases, which to some extent indicates improved discrimination between true DMP signals and background noise, thus maintaining a low false discovery proportion (FDP), defined as FP/(FP+TP). Beyond this q-value threshold, the difference between p-values and q-values narrows or becomes negligible, reducing the discriminability between true signals and background noise and increasing potential false positives. This method ensures that the q-value threshold is no longer an empirical value, but a data-driven adaptive value.

To verify the rationality of this q-value threshold (termed the adaptive q-value threshold, AQT), Supplementary Figure 3c above shows the values of evaluation metrics (Precision, Recall, F1, MCC) when q-value thresholds are set from 0.01 to 0.05, as well as for the AQT. The results show that the AQT generally corresponds to near-optimal detection performance.

Furthermore, we validated the difference between this adaptive threshold and the empirically set thresholds (0.035 for CHG, 0.049 for CHH) on the *Arabidopsis thaliana* dataset, as shown in Figure 1b (main text) and Supplementary Figure 2b and 3d–e. The AQT is approximately 0.000–0.004 lower for CHG and 0.005–0.009 lower for CHH than the empirical thresholds, indicating that the two values are relatively close, and the adaptive threshold is slightly more stringent for DMP filtering.

Notably, this maximum-difference-based adaptive thresholding strategy is a data-driven heuristic approach, rather than a threshold derived from strict statistical theory. Its rationality is generally consistent with available empirical observations. When combined with other complementary strategies (LowDiff-StrictVote and Adaptive Voting Threshold for DMPs, AVT-DMP), the integrated pipeline yields near-optimal detection metrics on simulated data with known ground truth, and produces conservative DMP sets with strong concordance with established tools on real biological datasets (Supplementary Figures 11g, 11j, 13, and 17).

In addition, we have made the q-value threshold customizable by users. MultiDMPcaller provides three options, ‑‑q‑cpg, ‑‑q‑chg, and ‑‑q‑chh, allowing users to specify q-value thresholds for the three methylation contexts (CpG, CHG, CHH) respectively; loci with q-values less than or equal to the threshold are included in the DMP set. If the user does not provide a custom q-value threshold, they must specify the '‑‑auto‑qvalue‑twostep' option, which allows the software to automatically determine the adaptive q-value threshold (AQT).

**Supplementary Figure 4**. Comparison of detection performance among the two-step p-value adjustment strategy adopted in this paper, the "Coverage prefilter + one-step FDR", "MethDiff prefilter + one-step FDR" and "One-step conventional correction (Standard FDR)" methods on the human simulated dataset.

**Note:** In our two‑step p‑value adjustment strategy, the raw p-value prefiltering step does not satisfy the independent filtering assumption required by the standard Benjamini-Hochberg (BH) procedure, since the same p-values are used for both pre-selection and subsequent multiple testing correction. To rigorously address this concern, we carried out three comparative experiments using simulated datasets with known ground truth, to evaluate both the overall detection performance of our method and its false-discovery control behavior.

We generated three human methylation simulated datasets corresponding to 2×2, 3×3, and 4×4 comparison designs using the pWGBSSimla module. The MultiDMPcaller analysis pipeline proceeds as follows: raw p-value prefiltering (p ≤ 0.05), BH correction on the filtered subset, and majority voting across all pairwise comparisons, with a stricter voting threshold specifically applied to loci with low absolute methylation differences (0–0.3). Instead of applying a hard cutoff to discard all low-difference loci, our method uses this graded voting strategy to retain biologically meaningful low-difference DMPs while controlling false positives. All results for our method in the subsequent comparisons are based on this workflow.

**1. Performance comparison under a uniform post-hoc filtering baseline**

To evaluate detection performance relative to conventional methods in methylome analysis workflows, we compared our full workflow with three representative reference approaches. For the three conventional methods, we applied a standard post-hoc filtering step that excludes all loci with absolute methylation difference ≤ 0.3 from their final outputs, which is a common practice in routine methylome data analysis. For our method, no such post-hoc hard cutoff was applied; instead, the aforementioned graded voting strategy for low-difference loci was used to retain only robustly supported DMPs in the final results.

The three reference strategies included in this comparison are:

**Coverage prefilter + one-step standard BH FDR**: Sites with coverage ≤ 15 are prefiltered prior to one-step BH correction, following the independent filtering rationale of Bourgon et al. (2010). This approach assumes conditional independence between site coverage and p-values from Fisher's exact test under the null hypothesis, thus preserving the theoretical validity of standard BH FDR control.

**MethDiff prefilter + one-step standard BH FDR**: Sites with absolute methylation difference ≤ 0.3 are prefiltered prior to one-step BH correction on the retained subset. Since absolute methylation difference and test p-values are not conditionally independent under the null hypothesis, this strategy does not satisfy the independent filtering assumption required for valid BH FDR control. Furthermore, as low-difference loci have already been excluded at the prefiltering stage, the aforementioned post-hoc filtering step has no additional effect on this approach.

**One-step standard BH FDR**: One-step BH correction applied directly to the full set of sites, as the conventional reference method.

As shown in Supplementary Figure 4, under this baseline setting, our pipeline achieves higher Recall, F1 score and MCC in most scenarios. Specifically, it outperforms the three reference methods by 1.17%–25.11% in Recall, 1.35%–17.38% in F1 score, and 0.86%–15.13% in MCC. The only exceptions occur under the 2×2 condition, where our method is 2.67% lower in F1 score and 3.63% lower in MCC than the conventional one-step method.

In terms of Precision, our method is approximately 1.58% to 18.5% lower than the reference methods. The gap is more pronounced under the 2×2 design with fewer replicates, and narrows gradually as the number of replicates increases, with nearly identical Precision under the 4×4 design.

This pattern is consistent with the difference in filtering strategies: conventional methods directly remove all loci with low absolute methylation differences to improve precision, while our method retains a subset of validated low-difference true DMPs through strict voting, resulting in a modest reduction in precision but consistently higher recall across all three replicate designs. Given the inherent precision-recall trade-off, our method achieves a more favorable balance between precision and recall under most conditions within this comparison framework.

Notably, negative loci (non-DMPs) account for the vast majority in the simulated dataset: true non-DMPs in the ground truth are approximately 35 to 41 times as abundant as true DMPs. As both Accuracy and Specificity are dominated by the majority negative class, these metrics consistently remain above 98% across all methods (Supplementary Figure 4), with differences less than 0.8% in all comparison settings.

**2. Performance comparison without post‑hoc filtering**

To directly assess whether our two-step p-value adjustment strategy inflates false discoveries relative to the standard BH procedure, we further compared our pipeline with the raw output of the conventional one-step standard BH method, with no post-hoc filtering of loci with absolute methylation differences ≤ 0.3. This comparison reflects the native false-discovery control behavior of each approach without additional post-processing.

As shown in Supplementary Figure 11g, on the 2×2 human dataset, the two‑step method achieves a lower false discovery proportion (FDP) and higher precision than the one‑step method, with a corresponding slight reduction in recall. Specifically, the two‑step method yields a precision of 65.32% and a recall of 72.59%, while the one‑step method achieves a precision of 51.47% and a recall of 74.59%.

This empirical evidence directly demonstrates that our two-step framework, paired with the majority voting mechanism across all pairwise comparisons, does not cause FDR inflation; instead, it provides more robust false-discovery control than the standard one-step BH procedure when loci with low absolute methylation differences are included.

**Supplementary Figure 5**. Proportion of false positives (yellow) detected for loci with low methylation difference levels on the 2×2 human simulated data, and the reduction in false positives after applying the 'LowDiff-StrictVote' strategy. The gray area represents the overlap between MultiDMPcaller detection results and ground truth DMPs (blue).

**Note:** **LowDiff-StrictVote strategy for false-discovery control**

Below, we elaborate on the strict high-voting-threshold strategy for low-methylation-difference loci, which is designed to reduce false positives. MultiDMPcaller provides the ‑‑dmp‑lowdiff‑strict‑vote option, which enables a strategy — abbreviated as LowDiff-StrictVote — that applies an elevated voting threshold to loci with low methylation differences. On human simulated data, we found that for loci with low absolute methylation difference *δ* (e.g., 0–0.3, 2823 loci in total), MultiDMPcaller was prone to classifying them as DMPs, resulting in high false positives (2441 loci, False Discovery Proportion (FDP) ≈ 86.47%, see Supplementary Figure 5a). A common feature of these false positive loci is that they often have insufficient voting support. For example, in 2×2 comparisons, most are supported 3 times (1776 loci, ~72.76% of false positive loci), while fewer have full 4-time support (665 loci). Therefore, for this subset of loci, our software provides a strategy to optionally increase the voting threshold for low-difference methylation loci to reduce false positives. When '--dmp-lowdiff-strict-vote ' is enabled with '--dmp-lowdiff-cutoff 0.3', the voting threshold for loci with absolute methylation differences of 0–0.3 is raised from the original majority support (e.g., 2/3) to a higher value *T*₁. For 2×2 comparisons, for example, this threshold corresponds to full support: a locus is included in the final DMP set only if all 4 individual comparisons support it as a DMP. *T*₁ is calculated as the ceiling of the mean of the total number of *m×n* pairwise comparisons and the voting threshold — either the user-defined *T*₀ or the software’s Adaptive Voting Threshold for DMPs (AVT-DMP). For example, in 3×3 comparisons (*m×n* = 9), if the software’s AVT is 6, then *T*₁ = (6 + 9)/2 = 7.5, rounded up to 8. We then sort the methylation differences from all *m×n* comparisons in descending order of their absolute values. The 'LowDiff-StrictVote' strategy is only applied if the *T*_0_-th value (for user-defined thresholds) or the AVT-DMP-th value (for adaptive thresholds) is ≤ 0.3.

The results show that this strategy effectively reduces false positives (as shown in Supplementary Figure 5b): the FDP decreases from 86.47% to 71.97%, a reduction of approximately 14.5 percentage points. Meanwhile, the overall FDP across all loci is approximately 34.7% (see Supplementary Figure 11g), indicating that low methylation difference intervals are prone to higher false positives.

To verify the rationality of this elevated adaptive threshold *T*₁, we calculated the values of each metric under three scenarios:

①no ‑‑dmp‑lowdiff‑strict‑vote option (lenient scenario),

②‑‑dmp‑lowdiff‑strict‑vote enabled with the elevated *T*₁ value (moderately stringent scenario), and

③‑‑dmp‑lowdiff‑strict‑vote enabled with unanimous support across all *m*×*n* comparisons (most stringent scenario).

We used the human 4×4 simulated dataset for this experiment to avoid the case where *T*₁ equals *m*×*n*. As shown in Supplementary Table 2 below, the second scenario — using the elevated threshold *T*₁ — achieves the best overall performance.

Alongside this strict voting threshold strategy, the absolute methylation difference threshold *δ* (e.g., 0.3) can be specified by the user via the ‑‑dmp‑lowdiff‑cutoff option, for example '--dmp-lowdiff-cutoff 0.3'.

Our two-step p-value adjustment strategy is restricted to specific cytosine sequence contexts: CHG and CHH in plants, and CpG in animals. For all other sequence contexts, DMP detection is performed using a conventional one-step FDR approach by default. Notably, however, when the `--dmp-lowdiff-strict-vote` option is enabled, the LowDiff-StrictVote strategy is applied to DMP detection across any species and all sequence contexts, as this helps reduce false positives among low-differential methylation loci.

**Supplementary Figure 6**. Modeling the distribution of DMP support counts on (a) the 2×2 and (b) the 4×4 human simulated datasets using the GMM-VT method, and identifying the cutoff between noise and signal. The adaptive voting threshold AVT-DMP is obtained by rounding up the cutoff value.

**Note:** On the human 4×4 simulated dataset, we tested multiple metrics under the adaptive voting threshold AVT-DMP and predefined voting thresholds round(4×4×*β*), where *β* = 1/16 (0.0625), 1/2 (0.5), 2/3 (0.66), 3/4 (0.75), and 1 (unanimous). We found that on this dataset, the 2/3 threshold — which matched the AVT-DMP — was an appropriate threshold, achieving balanced Precision and Recall as well as the highest F1 and MCC values (as shown in Supplementary Table 3 below).

To calculate the adaptive voting threshold AVT-DMP or AVT-DMR, we use the Bimodal Gaussian Mixture Model Valley Thresholding (GMM-VT) method to model the distribution of support counts for loci classified as DMPs or regions classified as DMRs, and identify the threshold that effectively distinguishes noise from signal as the optimal voting threshold. As shown in Supplementary Figure 6a, in 2×2 comparisons, we model the distribution of DMP support counts using an approximate bimodal normal distribution. It can be seen that DMPs supported 1 or 2 times are more abundant (considered background noise), while DMPs supported 3 or 4 times are significantly fewer (considered meaningful signals). There is a valley between the two peaks, which represents the optimal voting threshold; GMM-VT identifies this threshold via bimodal modeling. Loci with support counts ≥ this threshold are classified as DMPs. As shown in Supplementary Figure 6a and 6b, the AVT‑DMP thresholds are 3 for the 2×2 dataset and 11 for the 4×4 dataset, respectively.

The GMM-VT method is described as follows. Our basic assumption is that the distribution of locus support counts consists of two components: a low-support distribution representing noise or random background, and a high-support distribution representing true biological differences. Based on this, we fit the data using a bimodal Gaussian mixture model, which treats the overall data distribution as a weighted sum of two Gaussian distributions. Through iterative optimization via the Expectation-Maximization (EM) algorithm, the model automatically learns and fits the key parameters of the two Gaussian components, including their respective means, variances, and mixing weights. After the model converges successfully, the two Gaussian distribution curves intersect. Statistically, this intersection point (marked by the purple dashed line in Supplementary Figure 6 above) represents the position where the probability of a locus belonging to the noise component equals the probability of it belonging to the signal component. Therefore, we take the support count corresponding to this intersection point (rounded up) as the optimal voting threshold for distinguishing noise from signal. In rare cases where the data distribution is extremely abnormal and model fitting fails, the system defaults to the two-thirds majority voting threshold. The same method is used to determine the automatic voting threshold for DMRs (AVT-DMR).

**Supplementary Figure 7**. Identification of the adaptive DMR voting threshold AVT-DMR on the 2×2 human simulated dataset using the GMM-VT method (a special case where no regions are supported 1 or 2 times).

**Note:** It should be noted that, as shown in Supplementary Figure 7, in some special cases where support counts for the first half of *m×n* comparisons are all 0 (i.e., no DMRs are supported 1 or 2 times), our adaptive threshold determination program initially sets the threshold to 4. In this scenario, however, we consider 3 supports to also be valid, so the program automatically subtracts 1 from the initially determined optimal threshold (4 − 1 = 3), and uses 3 supports as the threshold for DMR classification.

**Supplementary Figure 8**. Genome-wide distribution of CpG DMPs, hyper-DMPs, hypo-DMPs, non-DMPs, hyper-DMRs, and hypo-DMRs across the five chromosomes of the *Arabidopsis thaliana* *epiF3* methylome.

**Supplementary Figure 9**. Association between methylation rate of the Arabidopsis chloroplast genome and sequencing coverage, and false methylation rate (FMR) profiling for sequencing quality validation.

**Note:** For the two sets of methylome datasets utilized in this study, namely: (1) Col-0_1, Col-0_2, *msh1_adv_1*, *msh1_adv_2*, *epiF3_1*, and *epiF3_2*; and (2) Wt_seg_1, Wt_seg_2, *msh1_gen1_1*, *msh1_gen1_2*, *msh1_gen2_dwf*_1, *msh1_gen2_dwf*_2, *msh1_gen2_var_1*, and *msh1_gen2_var_2*, we quantified and visualized the relationship between chloroplast methylation levels and sequencing coverage. This was performed following the method described by Becker *et al.* (2011) (see References) to demonstrate the high bisulfite conversion efficiency and reliable sequencing quality of our datasets.

(a, b): Two representative genotypes, *epiF3_1* and Wt_seg_1, were selected to illustrate the variations in chloroplast methylation levels. As sequencing coverage increases, the chloroplast methylation levels approach zero (black dots), and the average methylation level of these two genotypes remains close to zero across different coverage depths (red dots). Furthermore, loci with high sequencing coverage account for the vast majority of the total loci (indicated by the rightmost bars).

(c, d): Similarly, as coverage increases, the average chloroplast FMR for the first set of genotypes gradually decreases from approximately 40% or 20% to zero (panel c), whereas the average FMR for the second set remains consistently below 1.25% (panel d). Given that DNA methylation is virtually absent in plant chloroplasts, the low methylation levels observed at high sequencing coverage confirm the high quality of the methylome sequencing and the reliability of the generated data in this study.

**Supplementary Figure 10**. Relationship between methylation difference and DMP frequency in the 2×2 simulated human whole‑genome bisulfite sequencing (WGBS) data. The analyzed region corresponds to the 1,000,000–5,000,000 bp interval on chromosome 1.

**Note:** For the human simulated datasets, we used (*θ* = 0.6), under which a relatively large proportion of the methylation differences between the experimental and control groups were concentrated around 0.6. However, visualization of the methylation difference distribution showed that a considerable number of loci were also distributed across other ranges (Supplementary Figure 10); therefore, the concentration of differences around 0.6 was unlikely to substantially bias the overall conclusions presented in the main text. We additionally examined the (*θ* = 0.4) setting and found that most of the patterns and conclusions observed for the (*θ* = 0.6) datasets remained valid. Owing to space limitations, the results for (*θ* = 0.4) are not presented here.

**Supplementary Figure 11**. Overlap of DMPs detected by the two-step p-value adjustment method and the conventional one-step FDR method in human simulated and *Arabidopsis thaliana* real datasets.

(a–c) The relationship between the methylation differences of detected DMPs and the distribution of their occurrence counts in each individual comparison. The blue area denotes DMPs exclusively detected by the two-step method in each individual comparison, and the yellow area denotes DMPs shared by both methods. (a) Results for CpG-DMPs from the 2×2 human simulated dataset; (b–c) Results for CHG-DMPs and CHH-DMPs from the 2×2 real *Arabidopsis thaliana* dataset.

(d–f) The blue and yellow curves represent the distribution of methylation difference against locus count for **final DMPs** identified by the two-step method and the one-step method, respectively, across all individual comparisons. Consistent with the grouping above, (d) shows results for the human dataset, and (e–f) show results for *Arabidopsis thaliana*. It can be seen that on the human dataset, the DMP set yielded by the one-step method covers the vast majority of DMPs, whereas on the *Arabidopsis thaliana* dataset, the two-step method detects more DMPs than the one-step method.

(g–i) Show the overlap among DMP sets detected by the two-step method, the one-step method, and the ground truth. Similarly: (g) human simulated dataset; (h–i) real *Arabidopsis thaliana* dataset.

(j) Presents the overlap between DMP sets detected by our two-step method and those from other software tools on the Arabidopsis dataset.

**Note: Detailed false-discovery control comparison**

In the following, we elaborate on the differences in false-discovery control performance between our two-step strategy and the conventional one-step BH procedure.

In Supplementary Figures 11a–c, we found that in the 2×2 human simulated dataset, the number of CpG-DMPs exclusively detected by the two-step method was small, with a mean absolute methylation difference of approximately 0.2. By contrast, in the Arabidopsis dataset, the number of CHG-DMPs and CHH-DMPs uniquely identified by the two-step method was larger, with a mean absolute methylation difference of around 0.45.

Supplementary Figure 11d depicts the overlap of the **final DMP sets** yielded by the two-step method and the conventional one-step method on the human simulated dataset. As shown, on the human dataset, the one-step method recovers a greater number of DMPs but at a higher false discovery proportion (~48.5%; Supplementary Figure 11g), whereas the two-step method achieves a lower observed false discovery proportion (~34.7%; Supplementary Figure 11g). Supplementary Figures 11e-f illustrate the overlap of final CHG-DMPs and final CHH-DMPs between the two-step method and the one-step method in *Arabidopsis thaliana*, respectively. Remarkably, in the Arabidopsis dataset, the final DMP set from the two-step method fully encompasses that from the one-step method, and the two-step method additionally identifies a substantial number of DMPs that are not detected by the one-step method.

The contrasting patterns between Supplementary Figure 11d and 11e-f may be attributed to inherent differences in DNA methylation between animals and plants. For example, CpG methylation differences are relatively modest in animals, whereas CHG and CHH methylation differences are greater in plants (He et al., 2011). Since MultiDMPcaller applies a stricter high voting threshold to loci with low methylation differences (0–0.3) — for instance, requiring unanimous support across all 4 voting rounds — this results in higher precision but lower recall for the two-step method compared with the one-step method on the human dataset. Specifically, the two-step method achieves a precision of 65.32% and a recall of 72.59%, while the one-step method yields a precision of 51.47% and a recall of 74.59%.

Supplementary Figure 11g shows the overlap of DMPs among the two-step method, the one-step method and the ground truth on the human dataset, corroborating that the additional loci detected exclusively by the one-step method are largely false positives. Supplementary Figures 11h-i show the overlap between DMP sets detected by the two-step method and the one-step method on the Arabidopsis dataset. As no ground truth DMP set is available for this dataset, it is not included in the comparison. The results in Supplementary Figure 11j further demonstrate the degree of concordance between DMPs identified by the two-step method and those detected by other software tools on the Arabidopsis dataset. As can be seen, DMPs called by the two-step method constitute a subset of the outputs from other tools and cover the majority of the intersection of DMP sets from other tools, indicating the high conservativeness of the two-step method and suggesting a low false discovery proportion.

**Supplementary Figure 12**. Overlap of DMPs detected by the two-step p-value adjustment method and the conventional one-step FDR method in apple (Chr3, Chr5 and Chr10) and mouse (Chr7, Chr17 and Chr19) datasets.

(a–c) The relationship between the methylation differences of detected DMPs and the distribution of their occurrence counts in each individual comparison. The blue area denotes DMPs exclusively detected by the two-step method in each individual comparison, and the yellow area denotes DMPs shared by both methods. (a) Results for CpG-DMPs from the mouse dataset; (b–c) Results for CHG-DMPs and CHH-DMPs from the apple dataset. In each individual pairwise comparison, the number of DMPs unique to the two-step method is higher in apple than in mouse, consistent with the pattern in Supplementary Figures 11a-c.

(d–f) The blue and yellow curves represent the distribution of methylation difference against locus count for **final DMPs** identified by the two-step method and the one-step method, respectively, across all individual comparisons. Consistent with the grouping above, (d) shows results for the mouse dataset, and (e–f) show results for apple. In contrast to the human results shown in Supplementary Figure 11d, the two-step method yields more DMPs than the one-step method in the finalDMP dataset for mouse, and the same pattern is observed for apple.

(g–i) Show the overlap between DMP sets detected by the two-step and the one-step methods. Similarly: (g) mouse dataset; (h–i) apple datasets.

**Supplementary Figure 13**. Overlap of DMP detection results between MultiDMPcaller (two-step method) and other existing tools on (a) apple and (b) mouse methylome data.

**Note:** MultiDMPcaller uses the adaptive q-value threshold (‑‑auto‑qvalue‑twostep), adaptive voting threshold (‑‑auto‑dmp‑vote‑threshold), and the strict voting threshold strategy for low methylation difference loci (absolute methylation difference 0-0.3) (‑‑dmp‑lowdiff‑strict‑vote, ‑‑dmp‑lowdiff‑cutoff 0.3).

The reliability of our method on real biological datasets from two species: apple (Xu et al., 2018), and mouse (He et al., 2020) was further validated.

We downloaded mouse data from the study of He et al. (2020). Given that the mouse genome is large (~2.72 Gb, approximately 23 times that of *Arabidopsis thaliana* at 119 Mb), and to reduce computational cost, we selected three chromosomes with prominent methylation levels (Chr7/17/19) as the source data. As there is no ground truth for this dataset, we compared the results of MultiDMPcaller with those of other existing tools. Consistent with the high Precision and low false discovery proportion observed on the human simulated data (Supplementary Figure 17), the DMPs obtained by MultiDMPcaller’s two-step method form the smallest subset among all tools: MultiDMPcaller < DSS < MethylSig < methylKit (Supplementary Figure 13b).

Similarly, we selected three chromosomes with pronounced methylation levels (Chr3, Chr5 and Chr10) from the apple genome as the source dataset. As shown in Supplementary Figure 13a, 98%–100% of the DMPs identified by MultiDMPcaller are covered by the results of other existing tools in most scenarios; this proportion drops to 71% only for CHH-DMPs in the apple dataset (Supplementary Figure 13a, rightmost panel). Meanwhile, our calls also capture the vast majority of the consensus intersection across all compared tools. This overall pattern aligns with the conservative property of our method and suggests a potentially lower false discovery proportion (FDP) in real data, where no definitive ground truth is available.

Furthermore, we propose that the reduced overlap of CHH-DMPs in the apple dataset likely arises from two combined factors. First, the apple dataset adopts a 1×2 unbalanced design with only one control replicate and two treatment replicates. Conventional DMP detection tools such as methylKit and DSS rely on within-group biological variance estimation for statistical testing, which cannot be reliably performed with a single replicate per group. In contrast, MultiDMPcaller does not depend on within-group variance estimation, and thus its statistical behavior is less affected by the lack of replicates. Second, CHH methylation in plants exhibits higher stochastic variation and a lower signal-to-noise ratio across biological replicates compared with CpG and CHG methylation (Law and Jacobsen, 2010). The discrepancy in statistical frameworks between methods is disproportionately amplified in this high-noise, low-replicate scenario, leading to reduced concordance among detection call sets.

**Supplementary Figure 14**. Number of CG‑DMPs detected by MultiDMPcaller (without the LowDiff‑StrictVote+AQT+AVT‑DMP false‑discovery control strategy) and other tools, their overlap, and direction consistency of hyper- and hypo- methylation among overlapping DMPs in the Arabidopsis dataset. For overlap results with the strategy applied, see Supplementary Figure 11j.

**Supplementary Figure 15**. Overlap of CHG- and CHH-DMPs identified by MultiDMPcaller (without the LowDiff-StrictVote+AQT+AVT-DMP false-discovery control strategy) and two other tools (methylKit, DSS), and directional concordance of methylation changes (hyper- vs. hypo-methylation) in the Arabidopsis dataset. For overlap results with the strategy applied, see Supplementary Figure 11j.

**Supplementary Figure 16**. Overlap of DMRs identified by MultiDMPcaller and other software on the Arabidopsis dataset, and the directional concordance (i.e., hyper-methylation and hypo-methylation). (a) CpG context; other software includes DMRcaller, DMRcate, DSS, methylKit, and methylSig. (b) CHG and CHH contexts **without** false-discovery control strategies (LowDiff-StrictVote, AQT and AVT-DMP); other tools include DMRcaller, methylKit, and DSS. (c) CHG and CHH contexts **with** all three false-discovery control strategies applied.

**Note**: The number of CpG‑DMPs in the Arabidopsis dataset shows little overall change, as their detection is performed using the conventional one‑step FDR method rather than the two‑step procedure. The LowDiff‑StrictVote strategy alone only reduces the CpG‑DMP count by 527; therefore, the CpG‑DMR overlap results with other tools after application of the false‑discovery control strategy are not presented here.

**Supplementary Figure 17**. Performance comparison between MultiDMPcaller and existing tools across three human simulated datasets (*θ*=0.6). MultiDMPcaller achieves superior Precision, optimal F1 and MCC, and slightly lower Recall. Due to the large number of non-differentially methylated positions (Non-DMPs) and small number of DMPs, all tools achieve high Accuracy and Specificity (~98%), which are not shown here.

**Note:** On these three human simulated datasets corresponding to 2×2, 3×3, and 4×4 replicate designs, respectively, we evaluated the performance of our software MultiDMPcaller against widely used tools including methylKit, DSS, and MethylSig. As shown in Supplementary Figure 17, MultiDMPcaller achieves higher Precision than other tools in most cases (5.4%–54.3%), with the sole exception of the 3×3 comparison, where its Precision is 10.8% lower than MethylSig. For Recall, it is 1.1%–5.7% lower than other tools in most scenarios, and only 1.6%–23.3% higher than MethylSig. Given the inherent trade-off between Precision and Recall, F1 and MCC are metrics that evaluate overall balanced performance. Compared with other tools, MultiDMPcaller delivers the best F1 and MCC values (1%–36.7%). In addition, the large number of non-DMP loci (~90,000), which far outnumbers DMPs (~2,000), leads to nearly identical Accuracy and Specificity across all tools (around 98%), so these metrics are not shown in the figure.

**Supplementary Table 1**. Format example of the input files.

| Chromosome | Position | Methylated reads | Unmethylated reads | Methylation context |
| --- | --- | --- | --- | --- |
| chr3 | 5204618 | 0 | 1 | CHG |
| chr1 | 322644 | 0 | 4 | CHH |
| chr5 | 22472569 | 0 | 10 | CHG |
| chr1 | 5394291 | 0 | 2 | CHH |
| chr2 | 5042716 | 29 | 3 | CpG |
| chr4 | 6956544 | 17 | 3 | CpG |
| chr4 | 10461532 | 0 | 8 | CHH |
| chr1 | 9015129 | 24 | 3 | CHG |

**Supplementary Table 2**. Validation of the elevated voting threshold *T*₁ for low methylation difference scenarios.

| Methods | Accuracy(%) | Specificity(%) | Precision(%) | Recall(%) | F1(%) | MCC(%) |
| --- | --- | --- | --- | --- | --- | --- |
| No-LowDiff-StrictVote | 99.42 | 99.71 | 88.13 | **87.53** | 87.83 | 87.53 |
| LowDiff-StrictVote | **99.54** | 99.98 | 99.02 | 81.84 | **89.61** | **89.81** |
| LowDiff-UnanimousVote | 99.48 | **99.99** | **99.32** | 78.77 | 87.86 | 88.21 |

**Note**: On the 4×4 human simulated dataset, among the three scenarios — "no elevated threshold", "moderate elevated threshold *T*₁", and "unanimous vote" — the moderate threshold used by our software (determined as the ceiling of the average of the prior user-defined/adaptive voting threshold and the *m×n* total count) achieves balanced Precision and Recall, and the highest F1 and MCC scores.
**Supplementary Table 3**. Validation of the rationality of the adaptive voting threshold for DMPs (AVT-DMP).

| Voting threshold | Accuracy (%) | Specificity (%) | Precision (%) | Recall (%) | F1 (%) | MCC (%) | DMP count | DMR count |
| --- | --- | --- | --- | --- | --- | --- | --- | --- |
| round(4×4×*β*), *β*=1/16 | 58.06 | 57.19 | 5.11 | **93.63** | 9.69 | 15.67 | 40,592 | 24 |
| round(4×4×*β*), *β*=1/2 | 99.27 | 99.55 | 82.76 | 87.80 | 85.21 | 84.87 | 2,349 | 39 |
| round(4×4×*β*), *β*=2/3 | **99.54** | 99.98 | 99.02 | 81.84 | **89.61** | **89.81** | 1,830 | 39 |
| round(4×4×*β*), *β*=3/4 | 99.53 | 99.98 | 99.12 | 80.98 | 89.14 | 89.37 | 1,809 | 39 |
| round(4×4×*β*), *β*=1 | 99.02 | **100.00** | **99.77** | 59.30 | 74.39 | 76.54 | 1,316 | 36 |
| AVT-DMP | **99.54** | 99.98 | 99.02 | 81.84 | **89.61** | **89.81** | 1,830 | 39 |

**Note:** To verify the rationality of the adaptive voting threshold for DMPs (AVT-DMP) in the *m*×*n* comparison framework, we evaluated all metrics (Precision, Recall, etc.) under AVT and other thresholds set to round(*m*×*n*×*β*) on the human 4×4 simulated dataset (*m* = 4, *n* = 4). Here *β* takes values of 1/*k*, 1/2, 2/3, 3/4, and 1, with *k* = *m*×*n* = 16 for this dataset. A locus is classified as a final DMP if its DMP support count is ≥ round(*m*×*n*×*β*). As AVT-DMP equals round(4×4×(2/3)) on this dataset, the two rows highlighted in red have identical values.

**Supplementary Table 4**. Options and functional descriptions of MultiDMPcaller

| **Option** | **Description** |
| --- | --- |
| **‑‑auto‑qvalue‑twostep** | **Software-adaptive.** Automatically determined by the software. It derives the adaptive q-value threshold (AQT) by locating the point with the maximum difference between q-values and p-values. Note that AQT is only applicable to plant CHG/CHH contexts and animal CpG contexts. A detailed description is provided in Supplementary Figure 3. |
| **--auto-dmp-vote-threshold**  **--auto-dmr-vote-threshold** | **Software-adaptive.** The adaptive voting thresholds for DMPs (AVT-DMP) and DMRs (AVT-DMR) are derived via the bimodal Gaussian Mixture Model Valley Thresholding (GMM-VT) method, which models support count distributions to automatically determine the optimal signal-noise cutoff. Loci or regions with support counts greater than or equal to this threshold across all *m*×*n* pairwise comparisons are designated as final DMPs or DMRs, respectively (Supplementary Figures 6 and 7). |
| **‑‑q‑cpg** (default 0.05)  **‑‑q‑chg** (default 0.04)  **‑‑q‑chh** (default 0.045) | **User-defined**. Allows users to specify q-value thresholds for the three methylation contexts (CpG, CHG, CHH) respectively; loci with q-values below the threshold are classified as DMPs. To control false discovery, a threshold of < 0.04 is recommended for plant CHG/CHH and animal CpG contexts, while 0.05 is recommended for all other contexts. Alternatively, users may use the adaptive q-value threshold (AQT) automatically determined by the software via the --auto-qvalue-twostep option. |
| **‑‑dmr‑q** (default 0.05) | **User-defined**. The q-value threshold for inclusion in the final DMR set; regions with q-values ≤ this threshold are classified as DMRs. |
| **‑‑vote‑threshold** (default 2/3) | **User-defined**. Voting threshold applied across *m*×*n* pairwise comparisons, applicable to both DMP and DMR calling. A 2/3 majority support threshold is typical for *m*×*n* comparison designs. If no fixed value is specified, the software-adaptive AVT-DMP and AVT-DMR thresholds can be enabled via --auto-dmp-vote-threshold and --auto-dmr-vote-threshold respectively. |
| **‑‑dmp‑lowdiff‑strict‑vote**  **--dmp-lowdiff-cutoff** (default 0.3) | **User-defined**. If enabled, implements the LowDiff-StrictVote strategy, which applies an elevated voting threshold to loci with small methylation differences. The threshold value can be user-specified via the --dmp-lowdiff-cutoff option. See Supplementary Figure 5 for details. |
| **‑‑processes** (default 1) | **User-defined**. Number of worker processes allocated per job for parallel computing, defaulting to 1. MultiDMPcaller parallelizes independent computational steps, including *m*×*n* Fisher’s exact tests and preprocessing of *m*+*n* samples. For optimal efficiency, this value is recommended not to exceed the number of physical CPU cores on the server. |
| **‑‑methy‑diff‑dmp** (default 0)  **‑‑methy‑diff‑dmr** (default 0) | **User-defined**. Methylation difference thresholds *T*_diff_DMP_ and *T*_diff_DMR_ for DMPs and DMRs, respectively. The absolute methylation difference of identified DMPs and DMRs must meet or exceed their corresponding threshold. For example, in an individual comparison, a locus is deemed a DMP identified in this comparison if its q-value is ≤ the adaptive q-value threshold (AQT) or a user-specified threshold set via --q-cpg, --q-chg, or --q-chh, and its absolute methylation difference is ≥ *T*_diff_DMP_. If not specified, both thresholds default to 0. This type of filtering parameter is commonly used in existing comparable tools. |
| **‑‑dmr‑engine** (default python)**,**  **‑‑skip‑dmr,**  **‑‑skip‑window** | **User-defined**. Specifies which code implementation to use for DMR calling, and controls whether to skip DMR identification and whether to skip the visualization step. Since MultiDMPcaller required significant time to screen DMR candidate sets, we rewrote the source code for this functionality in C++ to further improve efficiency. Users can choose between the Python implementation (‑‑dmr‑engine python) and the C++ implementation (‑‑dmr‑engine cpp). Users may also choose to output only DMPs and skip DMR generation (‑‑skip‑dmr), or skip the DMP/DMR visualization step (‑‑skip‑window). |

**Supplementary Table 5**. Information on the final DMPs included in the result files (using a subset of CHG-context DMPs on Chromosome 1 of *Arabidopsis thaliana* as an example; only key columns are listed).

| Chromosome | Methylation_Type | Position | Methylation_Change^1^ | Hyper_Count^2^ | Hypo_Count^3^ | Num_Comparisons^4^ | Sig_Mean_Qvalue^5^ | qvalue_wt*i*_mut*j* ^6^ |
| --- | --- | --- | --- | --- | --- | --- | --- | --- |
| chr1 | CHG | 1703414 | 1 | 4 | 0 | 4 | 0.034761653 | 0.032318383 |
| chr1 | CHG | 2411844 | 1 | 4 | 0 | 4 | 0.020676183 | 0.029194488 |
| chr1 | CHG | 2411908 | 1 | 4 | 0 | 4 | 0.011249397 | 0.003600949 |
| chr1 | CHG | 4620473 | 0 | 0 | 4 | 4 | 0.016675705 | 0.002122175 |
| chr1 | CHG | 4620488 | 0 | 0 | 4 | 4 | 0.010102406 | 0.000169619 |
| chr1 | CHG | 4620554 | 0 | 0 | 4 | 4 | 0.000971586 | 0.000407379 |
| chr1 | CHG | 4830698 | 0 | 0 | 3 | 4 | 0.017165075 | 0.000906177 |
| chr1 | CHG | 4830726 | 0 | 0 | 3 | 4 | 0.009040012 | 6.49E-09 |

**Note**:

1: 1 indicates hyper-methylation, and 0 indicates hypo-methylation.

2：The number of times out of the *m* × *n* comparisons supporting the locus as a hyper-methylated DMP.

3：The number of times out of the *m* × *n* comparisons supporting the locus as a hypo-methylated DMP.

4：The total number of *m* × *n* comparisons. Note: (Hyper_Count + Hypo_Count) / Num_Comparisons ≥ the user-defined threshold or the adaptive voting threshold for DMPs (AVT-DMP).

5：The average q-value across the pairwise comparisons that support the locus as a DMP out of the *m* × *n* comparisons.

6：The q-value of the (*i*, *j*)-th comparison. Note: (1) *i* = 1...*m*, *j* = 1...*n*. Here, we only present the data for *i* = 1 and *j* = 1. (2) For CHG and CHH contexts in plants utilizing the two-step p-value adjustment strategy, loci with a raw p-value > 0.05 are excluded from the FDR correction and thus cannot be classified as DMPs; their q-values are uniformly set to 1.

**Supplementary Table 6**. Information on the final DMRs included in the result files (using a subset of CpG-context DMRs on Chromosome 1 of *Arabidopsis thaliana* as an example; only key columns are listed).

| Chromosome | Methylation_Type | DMR_start | DMR_end | Length | Direction^1^ | Sig_count^2^ | Total_count^3^ | Sig_Avg_qvalue^4^ | qvalue_mut*i*_wt*j* ^5^ |
| --- | --- | --- | --- | --- | --- | --- | --- | --- | --- |
| chr1 | CpG | 3984601 | 3986600 | 2000 | 0 | 4 | 4 | 1.59E-28 | 1.00E-33 |
| chr1 | CpG | 4122201 | 4125600 | 3400 | 1 | 3 | 4 | 0.001868069 | 2.74E-05 |
| chr1 | CpG | 4175101 | 4177600 | 2500 | 1 | 4 | 4 | 7.38E-16 | 2.23E-19 |
| chr1 | CpG | 4178601 | 4181200 | 2600 | 0 | 4 | 4 | 3.85E-06 | 1.38E-05 |
| chr1 | CpG | 4368101 | 4370300 | 2200 | 1 | 4 | 4 | 1.39E-11 | 6.25E-84 |
| chr1 | CpG | 5564101 | 5568500 | 4400 | 0 | 3 | 4 | 0.02360543 | 0.394249 |
| chr1 | CpG | 5749801 | 5752100 | 2300 | 1 | 3 | 4 | 0.010633037 | 0.028685 |

**Note**:

1: 1 indicates hyper-methylation, and 0 indicates hypo-methylation.

2：The number of times out of the *m* × *n* comparisons supporting the region as a DMR.

3：The total number of *m* × *n* comparisons. Note: Sig_count / Total_count ≥ the user-defined threshold or the adaptive voting threshold for DMRs (AVT-DMR).

4：The average q-value across the pairwise comparisons that support the region as a DMR out of the *m* × *n* comparisons.

5：The q-value of the (*i*, *j*)-th comparison, where *i* = 1...*m* and *j* = 1...*n*. Here, we only present the data for *i* = 1 and *j* = 1.

**Supplementary Table 7**. Versions, parameters, preprocessing pipelines, and execution commands of compared software

| **Software and Exact Version** | **Parameter Settings** | **Data Preprocessing Pipeline** | **Execution Command** |
| --- | --- | --- | --- |
| MultiDMPcaller 1.0.0 | *m* × *n* pairwise Fisher exact tests; animal mode (--biotype 0); --auto-qvalue-twostep; --auto-dmp-vote-threshold; --dmp-lowdiff-strict-vote; --dmp-lowdiff-cutoff 0.3; --processes 8; --skip-dmr; --skip-window. | Use the five-column CpG read-count tables (chromosome, position, methylated reads, unmethylated reads and context). WT and treatment replicates are supplied in separate directories. No additional coverage cutoff was specified in the benchmark command. Notably, the built-in data preprocessing module of the software automatically removes loci with coverage < 4. | python -u MultiDMPcaller_final_code_English_version.py \  --wt-reps 2 \  --mut-reps 2 \  --dir-wt ./wt_data_theta0.6_rep2×2 \  --dir-mut ./mut_data_theta0.6_rep2×2 \  --biotype 0 \  --auto-qvalue-twostep \  --auto-dmp-vote-threshold \  --dmp-lowdiff-strict-vote \  --dmp-lowdiff-cutoff 0.3 \  --processes 8 \  --skip-dmr \  --skip-window |
| methylKit 1.36.0 | pipeline="amp"; methRead mincov=10; unite(..., destrand=FALSE); calculate DiffMeth using the methylKit package-default statistical settings, with mc.cores=4; getMethylDiff(..., qvalue=0.05, difference=20). Final filtering: q-value ≤ 0.05 and \|meth.diff\| ≥ 20 percentage points. | Convert the cleaned five-column methylation count files into methylKit AMP-format files while retaining CpG records. Import the AMP files using methRead with mincov=10. No additional filterByCoverage step was applied. The retained loci were united across all samples using destrand=FALSE before differential methylation analysis. | Rscript run_methylkit_one_cleaned_CpG.R \  theta0.6_rep2×2 2 4 20 |
| DSS 2.58.0; R 4.5.3 | DSS 2.58.0 under R 4.5.3; DSS differential methylation model; smoothing=FALSE; FDR cutoff=0.05; use the \|Delta methylation\|>=0.20 output for direct comparison; all biological replicates included. | Convert the same methylated/unmethylated CpG read-count files into BSseq-compatible input. | Rscript DSS/benchmark_run_one_dss.R 0.6 2×2 \  ./theta0.6_rep2×2/output |
| MethylSig 1.22.0; R 4.5.3;  Bioconductor 3.22 | diff_methylsig; FDR ≤ 0.05; \|meth_diff\| ≥ 20%; disp_groups=c(case=TRUE, control=TRUE); local_window_size=0; t_approx=TRUE; n_cores=4 | Read the five-column count files and retain valid CpG/CG records. Aggregate duplicated chromosome-position records by summing the methylated and unmethylated read counts, calculate coverage, and retain records with coverage > 0. Construct a BSseq object from the aligned count matrices. Before differential testing, require each retained locus to have non-zero coverage in all biological replicates of both the control and treatment groups. | Rscript scripts/run_methylsig_cpg.R theta0.6_rep2×2 \  ./datasets_clean_pairmean001 \  ./methylSig_theta0.6_rep2×2 4 |

**Note**: The R/Bioconductor packages methylKit, DSS, and MethylSig are not standalone command-line tools that execute a full workflow with a single command like MultiDMPcaller. The listed Rscript commands represent the actual shell-level execution calls; the package-specific function calls and arguments are defined inside the corresponding R scripts. The complete executable scripts—including input preprocessing procedures, parameter configurations, parallelization settings, final output-filtering criteria, and example commands for the 2×2, 3×3, and 4×4 replicate designs—are permanently archived at Zenodo (<https://doi.org/10.5281/zenodo.21717274>).

As shown above, we provided the exact version numbers, parameter settings, data preprocessing pipelines, and shell-level execution commands of all compared software in Supplementary Table 7. The comparison was performed on the human 2×2 simulated dataset (two replicates in the experimental group, two replicates in the control group) with θ=0.6, meaning the absolute methylation difference between the experimental and control groups was preset to 0.6. A small fraction of loci with methylation differences of 0–0.01 (accounting for approximately 0.01%–0.08% of total loci) were treated as noise and removed from the generated simulated dataset. Such extremely small between-group methylation differences are generally undetectable by standard DMP calling tools, so their exclusion allows for more accurate calculation of performance evaluation metrics.

Notably, to unify the significance threshold across all tools, we applied an FDR/q-value cutoff of ≤ 0.05 for methylKit, DSS, and MethylSig, whereas MultiDMPcaller used an adaptive q-value threshold (AQT) based on its two-step adjustment strategy.

For methylation difference filtering, all compared tools except MultiDMPcaller applied a uniform hard cutoff: only loci with an absolute methylation difference ≥ 0.2 were retained. This post-filtering step was not used for MultiDMPcaller, as it would eliminate the core advantage of its built-in graded voting framework: an adaptive voting threshold for DMPs (AVT-DMP) across all *m*×*n* pairwise comparisons (2×2 in this case), with an even stricter unanimous-support rule for candidate DMPs with an absolute methylation difference ≤ 0.30.

The same analysis pipeline applies to the 3×3 and 4×4 datasets and is not described separately.

**Supplementary Table 8**. Computational resource consumption and runtime performance of MultiDMPcaller and other compared tools under a uniform computing environment

| **Software & Dataset** | **CPU Hardware & Core Allocation** | **Average CPU Utilization** | **Memory & Operating System** | **Parallel Configuration** | **Peak Memory Usage** | **Runtime** | **Output File Size** |
| --- | --- | --- | --- | --- | --- | --- | --- |
| MultiDMPcaller 1.0.0 (2×2) | Intel Xeon E5-2680 v2 @ 2.80 GHz; host 40 logical CPUs; 10 CPUs available to each benchmark job | 302% (~3.02 cores) | 64 GiB; Ubuntu 22.04 LTS | 8 workers | 0.60 GiB | 229.82 s | 0.331 MB (final CpG DMP file) |
| methylKit 1.36.0 (2×2) | Intel Xeon E5-2680 v2 @ 2.80 GHz; host 40 logical CPUs; 10 CPUs available to each benchmark job | 276% (~2.76 cores) | 64 GiB; Ubuntu 22.04 LTS | 4 threads | 1.13 GiB | 89.75 s | 0.505 MB (benchmark-recorded output) |
| DSS 2.58.0 (2×2) | Intel Xeon E5-2680 v2 @ 2.80 GHz; host 40 logical CPUs; 10 CPUs available to each benchmark job | 275% (~2.75 cores) | 64 GiB; Ubuntu 22.04 LTS | No explicit DSS thread option; job cap=10 | 1.17 GiB | 37.60 s | 19.511 MB (benchmark output directory) |
| MethylSig 1.22.0 (2×2) | Intel Xeon E5-2680 v2 @ 2.80 GHz; host 40 logical CPUs; 10 CPUs available to each benchmark job | 283% (~2.83 cores) | 64 GiB; Ubuntu 22.04 LTS | n_cores=4; BLAS/OpenMP=1 | 1.14 GiB | 64.48 s | 112 B (final result file; no significant DMP) |
| MultiDMPcaller 1.0.0 (3×3) | Intel Xeon E5-2680 v2 @ 2.80 GHz; host 40 logical CPUs; 10 CPUs available to each benchmark job | 332% (~3.32 cores) | 64 GiB; Ubuntu 22.04 LTS | 8 workers | 0.72 GiB | 486.04 s | 0.405 MB (final CpG DMP file) |
| methylKit 1.36.0 (3×3) | Intel Xeon E5-2680 v2 @ 2.80 GHz; host 40 logical CPUs; 10 CPUs available to each benchmark job | 283% (~2.83 cores) | 64 GiB; Ubuntu 22.04 LTS | 4 threads | 1.14 GiB | 83.56 s | 0.274 MB (benchmark-recorded output) |
| DSS 2.58.0 (3×3) | Intel Xeon E5-2680 v2 @ 2.80 GHz; host 40 logical CPUs; 10 CPUs available to each benchmark job | 269% (~2.69 cores) | 64 GiB; Ubuntu 22.04 LTS | No explicit DSS thread option; job cap=10 | 1.19 GiB | 39.00 s | 18.573 MB (benchmark output directory) |
| MethylSig 1.22.0 (3×3) | Intel Xeon E5-2680 v2 @ 2.80 GHz; host 40 logical CPUs; 10 CPUs available to each benchmark job | 281% (~2.81 cores) | 64 GiB; Ubuntu 22.04 LTS | n_cores=4; BLAS/OpenMP=1 | 1.15 GiB | 75.67 s | 0.151 MB (final result file) |
| MultiDMPcaller 1.0.0 (4×4) | Intel Xeon E5-2680 v2 @ 2.80 GHz; host 40 logical CPUs; 10 CPUs available to each benchmark job | 470% (~4.70 cores) | 64 GiB; Ubuntu 22.04 LTS | 8 workers | 1.29 GiB | 606.50 s | 0.696 MB (final CpG DMP file) |
| methylKit 1.36.0 (4×4) | Intel Xeon E5-2680 v2 @ 2.80 GHz; host 40 logical CPUs; 10 CPUs available to each benchmark job | 267% (~2.67 cores) | 64 GiB; Ubuntu 22.04 LTS | 4 threads | 1.15 GiB | 90.32 s | 0.191 MB (benchmark-recorded output) |
| DSS 2.58.0 (4×4) | Intel Xeon E5-2680 v2 @ 2.80 GHz; host 40 logical CPUs; 10 CPUs available to each benchmark job | 284% (~2.84 cores) | 64 GiB; Ubuntu 22.04 LTS | No explicit DSS thread option; job cap=10 | 1.18 GiB | 42.10 s | 18.601 MB (benchmark output directory) |
| MethylSig 1.22.0 (4×4) | Intel Xeon E5-2680 v2 @ 2.80 GHz; host 40 logical CPUs; 10 CPUs available to each benchmark job | 289% (~2.89 cores) | 64 GiB; Ubuntu 22.04 LTS | n_cores=4; BLAS/OpenMP=1 | 1.16 GiB | 79.71 s | 0.269 MB (final result file) |

Note: The output file organization varies across tools, so the measured scope for file size is specified for each entry in the "Output file size" column. MethylSig (2×2) detected no significant DMPs at FDR = 0.05, so the final result file contains only the header.

**Additional Note**: We present the computational resource consumption of all tools including MultiDMPcaller in Supplementary Table 8, covering CPU hardware and core allocation, average CPU utilization, memory and operating system, number of parallel execution units (parallelism type: multithreading for other tools, multiprocessing for MultiDMPcaller), peak memory usage, runtime, and output file size. The results show that MultiDMPcaller has comparable computational resource consumption to other tools, but its runtime is relatively longer, approximately 2.6–14.4 times that of other tools. This is mainly attributed to the *m*×*n* pairwise cross-group comparisons required by the method, as well as subsequent steps including adaptive q-value threshold estimation, adaptive voting threshold determination, and strict voting filtering for low-difference loci.

Notably, MultiDMPcaller implements parallel processing for both *m*×*n* pairwise comparisons and preprocessing across *m*+*n* samples. This parallelization is built on Python's multiprocessing pool technology and operates at the process level, whereas most other compared tools support thread-level parallelization. Accordingly, the parallel configuration option is designated as `--processes` for our software, and `--threads` for the other tools; based on the available CPU resources in the computing environment, these values were set to 8 and 4 respectively.

**Supplementary Table 9**. Scalability of MultiDMPcaller across different data scales and replicate designs

| **Dataset** | **Species & Data Type** | **Analysis Scope** | **Replicate Design (No. of Comparisons)** | **Total Input Loci (Cumulative Across Samples)** | **Average Input Loci Per Sample** | **Average Sequencing Depth (×)** | **Parallel Configuration (Workers / Threads)** | **Runtime (h)** | **Peak Memory Usage (GiB)** | **Total DMPs** | **Total DMRs** |
| --- | --- | --- | --- | --- | --- | --- | --- | --- | --- | --- | --- |
| Human simulated *θ*=0.6 | Human, simulation | Simulated region | 2×2 (4) | 372,076 | 93,019 | 47.34 | 10 | 0.07 | 0.60 | 2,895 | 56 |
| Human simulated *θ*=0.6 | Human, simulation | Simulated region | 3×3 (9) | 555,390 | 92,565 | 47.39 | 10 | 0.10 | 0.72 | 1,864 | 43 |
| Human simulated *θ*=0.6 | Human, simulation | Simulated region | 4×4 (16) | 737,480 | 92,185 | 47.44 | 10 | 0.17 | 1.30 | 1,830 | 39 |
| Arabidopsis whole genome | Arabidopsis | Chr1~Chr5 | 2×2 (4) | 89,656,861 | 22,414,215 | 8.91 | 10 | 1.75 | 7.25 | 31,758 | 344 |
| Mouse 3 chromosomes | Mouse | Chr7/17/19 | 2×2 (4) | 20,289,830 | 5,072,458 | 14.61 | 10 | 3.13 | 8.76 | 309,765 | 4,287 |
| Apple 3 chromosomes | Apple | Chr3/5/10 | 1×2 (2) | 118,268,415 | 39,422,805 | 14.11 | 10 | 5.90 | 11.16 | 259,507 | 4,646 |
| Apple all chromosomes | Apple | Chr00~Chr17 | 1×2 (2) | 637,062,140 | 212,354,047 | 13.53 | 10 | 34.89 | 43.13 | 1,321,457 | 22,448 |

Notes:
1. The analyzed region in the human simulated datasets corresponds to the 1,000,000–5,000,000 bp interval on Chr1.
2. Total input loci refers to the cumulative count of valid methylated loci (with non-zero coverage) across all samples; average input loci per sample is calculated by dividing the cumulative count by the number of samples. The same genomic position is counted separately in different samples.
3. Average sequencing depth = sum of coverage depths of all valid cytosine loci across all samples ÷ total number of valid cytosine loci. The coverage depth of a single cytosine locus = number of methylated reads + number of unmethylated reads at that locus; valid loci are defined as loci with non-zero coverage.

**Additional Note**: Supplementary Table 9 summarizes the detection results of MultiDMPcaller on human simulated (Chung and Kang, 2020), Arabidopsis (Virdi et al., 2015), mouse (He et al., 2020), and apple (Xu et al., 2018) datasets. The table lists chromosomes included, number of biological replicates, total and average input loci, sequencing depth, number of parallel processes/threads, runtime, peak memory usage, and counts of DMPs/DMRs for each dataset. The results demonstrate that MultiDMPcaller has favorable scalability and adaptability across different scenarios. Specifically, for the simulated datasets with biological replicates increasing from 2×2 to 3×3 and 4×4, all runtime metrics of MultiDMPcaller remain within a reasonable range. For the apple dataset, runtime metrics scale proportionally when expanding from 3 chromosomes to the full set of chromosomes, with complete output results.

**Supplementary Table 10**. Validation of the association between methylation changes (hyper-/hypo-methylation) and small RNA (sRNA) cluster expression changes (up-/down-regulation) via Support Vector Machine (SVM) on the *Arabidopsis thaliana* dataset.

| Screening criteria | Number of differentially expressed sRNA clusters | | Number and proportion of DMPs | | | Leave-one-out cross-validation accuracy (feature extraction applied, top 10 important features retained) | |
| --- | --- | --- | --- | --- | --- | --- | --- |
| sRNA clusters containing at least 1 DMP | **up**-regulated | 81 | **hyper**-methylated | **214** | **82%** | Linear kernel | 75.3 |
|  |  |  | hypo-methylated | 47 | 18% |  |  |
|  | **down**-regulated | 81 | hyper-methylated | 40 | 10.1% | RBF kernel | 77.8% |
|  |  |  | **hypo**-methylated | **356** | **89.9%** |  |  |
| sRNA clusters containing at least 3 DMPs | **up**-regulated | 28 | **hyper**-methylated | **170** | **87.6%** | Linear kernel | 89.3% |
|  |  |  | hypo-methylated | 24 | 12.4% |  |  |
|  | **down**-regulated | 28 | hyper-methylated | 22 | 6.6% | RBF kernel | 92.9% |
|  |  |  | **hypo**-methylated | **309** | **93.4%** |  |  |

**Note**: DMPs include all three contexts: CpG, CHG, and CHH. To ensure the Support Vector Machine (SVM) model can be trained and evaluated on a balanced dataset of positive and negative samples, we selected an equal number of up-regulated and down-regulated small RNA clusters. Upstream analysis of the small RNA-seq data was performed using the ShortStack tool (Axtell *et al.*, 2013). For downstream analysis, the chi-square test and Fisher's exact test were utilized to identify regions with a q-value < 0.05, which were then defined as significantly differentially expressed small RNA clusters.

In the comparison of *epiF3* vs Col-0, the association between methylation alterations and sRNA cluster expression changes can be effectively captured by the machine learning approach, yielding a cross-validation accuracy of approximately 77.8% or 92.9%. Statistical analysis revealed that for up-regulated sRNA clusters, the vast majority of DMPs (approximately 82% or 87.6%) were hyper-methylated, whereas for down-regulated sRNA clusters, the vast majority of DMPs (approximately 89.9% or 93.4%) were hypo-methylated. This indicates a strong positive correlation between the two molecular events, which aligns with the high accuracy achieved by the machine learning method.

The implementation of the machine learning method primarily comprises three components: feature construction, class label assignment, and model construction with cross-validation.

(1) Feature Construction

We extracted the following metrics from the DMPs within each differentially expressed sRNA cluster to serve as features (only key features are listed below). These individual features were concatenated to form a comprehensive feature vector. Zero-padding was applied to samples with insufficient feature dimensions. The features are categorized into two groups: "features of individual DMPs" and "aggregate features of all DMPs within the sRNA cluster."

(i) Features of individual DMPs

- Difference in methylation ratio, defined as *a*/(*a*+*b*) - *c*/(*c*+*d*), where (*a*, *b*) and (*c*, *d*) represent the (methylated read count, unmethylated read count) for the experimental and control groups, respectively. The difference in methylation ratio from each of the *m* × *n* pairwise comparisons (2 × 2 comparisons in our dataset) is provided sequentially; the same principle applies below.
- Methylation change direction of the DMP: 1 for hyper-methylation, 0 for hypo-methylation (*m* × *n* values in total).
- -ln(q-value), representing the significance level of the differential methylation (*m* × *n* values in total).

(ii) Aggregate features of all DMPs within the sRNA cluster

- Number of hyper-methylated DMPs contained within the sRNA cluster.
- Number of hypo-methylated DMPs contained within the sRNA cluster.
- Sum of methylated and unmethylated read counts across all DMPs within the sRNA cluster.
- Difference in methylation ratio calculated based on the total methylated and unmethylated read counts of all DMPs within the sRNA cluster (*m* × *n* values in total).

(2) Class Label Assignment

1：Positive class, up-regulated sRNA clusters.

-1：Negative class, down-regulated sRNA clusters.

(3) Model Construction with Cross-Validation

Both linear and radial basis function (RBF) kernels (with default parameters) were applied independently for model training and prediction using leave-one-out cross-validation (LOOCV). In brief, one sample was reserved as the test set in each iteration and the remaining samples formed the training set. This procedure was repeated for a number of iterations equal to the total sample count, and the average prediction accuracy across all iterations was reported as the final performance metric.

**Supplementary Table 11**. Association between hyper-/hypo-methylated DMPs and differential expression of small RNA clusters in apple.

| Screening criteria | Number of differentially expressed sRNA clusters | | Number and proportion of DMPs | | | Leave-one-out cross- validation accuracy | |
| --- | --- | --- | --- | --- | --- | --- | --- |
| sRNA clusters containing at least 1 DMP | **up**-regulated | 10 | **hyper-** methylated | **79** | **89.8%** | Linear kernel | 83.3% |
|  |  |  | hypo-methylated | 9 | 10.2% |  |  |
|  | **down**-regulated | 38 | **hyper-** methylated | **75** | **53.2%** | RBF kernel | 83.3% |
|  |  |  | hypo-methylated | 66 | 46.8% |  |  |
| sRNA clusters containing at least 3 DMPs | **up**-regulated | 4 | **hyper-** methylated | **72** | **93.5%** | Linear kernel | 92.0% |
|  |  |  | hypo-methylated | 5 | 6.5% |  |  |
|  | **down**-regulated | 21 | **hyper-** methylated | **68** | **57.1%** | RBF kernel | 92.0% |
|  |  |  | hypo-methylated | 51 | 42.9% |  |  |

**Note:** To further validate the biological relevance of the identified DMPs (as demonstrated in Supplementary Table 10 for *Arabidopsis thaliana*), we performed additional analysis using treatment (drought-tolerant) and control groups of Qinguan apple (Xu et al., 2018; Niu et al., 2018). Specifically, we used MultiDMPcaller to call DMPs across all apple chromosomes, and identified differentially expressed small RNA clusters following the method described in Supplementary Table 10.

As shown in Supplementary Table 11, within up-regulated differentially expressed small RNA clusters, hyper-methylated DMPs still account for the majority (89.8%–93.5%). Within down-regulated clusters, hyper- and hypo-methylated DMPs each account for approximately half (53.2%–57.1% and 42.9%–46.8%, respectively). Although this positive correlation is not as strong as that observed in *Arabidopsis thaliana*, support vector machine (SVM) machine learning still achieves high leave-one-out cross-validation accuracy (83.3%–92.0%). Given the straightforward feature encoding scheme, which consists primarily of DMP hyper-methylation (encoded as 1) and hypo-methylation (encoded as 0), linear and RBF kernels yield identical classification accuracy. This indicates that MultiDMPcaller can obtain robust DMPs in plant species other than *Arabidopsis thaliana*, and the results can be associated with other omics data, which is of certain biological significance.

**References**

Axtell MJ. ShortStack: comprehensive annotation and quantification of small RNA genes. RNA 2013;19:740–751.

Becker C, Hagmann J, Müller J et al. Spontaneous epigenetic variation in the Arabidopsis thaliana methylome. Nature 2011;480:245–249.

Bourgon R, Gentleman R, Huber W. Independent filtering increases detection power for high-throughput experiments. Proc Natl Acad Sci USA 2010;107:9546–9551.

Chung RH, Kang CY. pWGBSSimla: a profile-based whole-genome bisulfite sequencing data simulator incorporating methylation QTLs, allele-specific methylations and differentially methylated regions. Bioinformatics 2020;36:660–665.

He XJ, Chen T, Zhu JK. Regulation and function of DNA methylation in plants and animals. Cell Res 2011;21:442–465.

He Y, Hariharan M, Gorkin DU et al. Spatiotemporal DNA methylome dynamics of the developing mouse fetus. Nature 2020;583:752–759.

Law JA, Jacobsen SE. Establishing, maintaining and modifying DNA methylation patterns in plants and animals. Nat Rev Genet 2010;11:204–220.

Niu C, Li H, Jiang L et al. Genome-wide identification of drought-responsive microRNAs in two sets of Malus from interspecific hybrid progenies. Hortic Res 2019;6:75.

Virdi KS, Laurie JD, Xu YZ et al. Arabidopsis MSH1 mutation alters the epigenome and produces heritable changes in plant growth. Nat Commun 2015;6:6386.

Xu J, Zhou S, Gong X et al. Single-base methylome analysis reveals dynamic epigenomic differences associated with water deficit in apple. Plant Biotechnol J 2018;16:672–687.
